# Factors Associated With Pediatric Health Care Professionals’ Intention to Adopt Socially Assistive Robots: Mixed Methods Study

**DOI:** 10.2196/105501

**Published:** 2026-10-15

**Authors:** Yun-Hsuan Lee, Fang-Yu Hsu, Sue-hsien Chen, Shih-Ming Chu, Chih-Yuan Yang, Feng-Hsuan Liu, Angela Shin-Yu Lien

**Affiliations:** 1 Graduate Institute of Nursing, College of Medicine, Chang Gung University, Taoyuan, Taiwan; 2 Department of Nursing, Linkou Chang Gung Memorial Hospital, Taoyuan, Taiwan; 3 Department of Nursing Management, Chang Gung Medical Foundation Administration, Taoyuan, Taiwan; 4 School of Nursing, College of Medicine, Chang Gung University, Guishan, 259, Wenhua 1st Road, Taoyuan, 33375, Taiwan, 886 03-2118800 ext 5186; 5 Department of Pediatrics, Chang Gung Memorial Hospital, Taoyuan, Taiwan; 6 Department of Artificial Intelligence, Chang Gung University, Taoyuan, Taiwan; 7 Artificial Intelligence Research Center, Chang Gung University, Taoyuan, Taiwan; 8 Department of Internal Medicine, Division of Endocrinology and Metabolism, Linkou Chang Gung Memorial Hospital, Taoyuan, Taiwan; 9 Department of Endocrinology and Metabolism, Linkou Chang Gung Memorial Hospital, Taoyuan, Taiwan

**Keywords:** socially assistive robots, technology acceptance, health care professionals, pediatric nursing, UTAUT2, mixed methods, behavioral intention, human-robot interaction, Unified Theory of Acceptance and Use of Technology 2

## Abstract

**Background:**

Socially assistive robots (SARs) have the potential to support pediatric care; yet, routine clinical adoption remains limited. Because SARs interact directly with children, health care professionals (HCPs) may consider not only clinical utility and usability but also child-robot interaction, trust, and implementation concerns that may not be fully represented by conventional technology acceptance frameworks.

**Objective:**

This study aimed to examine factors associated with pediatric HCPs’ behavioral intention (BI) to adopt SARs and explored how clinical perspectives contextualized conventional technology acceptance constructs.

**Methods:**

A convergent parallel mixed methods study was conducted at a tertiary medical center in Taiwan between June and December 2025. In the quantitative strand, 125 pediatric HCPs viewed a standardized video of a SAR interacting with a child and completed a Unified Theory of Acceptance and Use of Technology (UTAUT2)-based questionnaire incorporating trust. Multiple linear regression examined associations with BI. In the qualitative strand, 17 HCPs with direct clinical SAR exposure, recruited primarily from 2 pilot units, participated in semistructured interviews. The strands involved partially overlapping but nonequivalent samples with different levels of SAR exposure. Qualitative data were analyzed thematically, and findings were integrated using a joint display.

**Results:**

The quantitative model accounted for 72.7% of the variance in BI. Social influence (SI: β=0.348; *P*<.001), hedonic motivation (HM: β=0.307; *P*<.001), and performance expectancy (PE: β=0.238; *P*=.001) had the largest standardized coefficients; the association for trust was small and sensitive to model specification, whereas effort expectancy (EE) and facilitating conditions (FC) were not significantly associated with BI. Limited discriminant validity and potential common method variance warranted caution in construct-specific interpretation. Qualitative analysis generated 6 themes concerning children’s emotional and stress-related challenges, SAR functions and clinical positioning, HCP attitudes, child-centered design, and implementation and institutional considerations. Integration showed convergence for clinical usefulness and complementarity for trust-related concerns. Children’s engagement and relational interaction were conceptually distinct from UTAUT2-defined HM, reflecting HCPs’ own enjoyment. SI was prominent quantitatively but was not articulated as a salient consideration in participants’ accounts, whereas operational concerns were elaborated in detail in participants’ accounts despite the nonsignificant association for EE.

**Conclusions:**

Pediatric HCPs’ intentions to adopt SARs were associated with SI, HM, and PE, but measurement limitations and the predominantly video-based evaluation context preclude interpreting these associations as distinct determinants or predictors of actual use. Qualitative findings highlighted child-centered relational value and context-specific implementation concerns. Child-centered relational value was conceptually distinct from HCP-centered HM, suggesting that evaluations of patient-facing socially interactive technologies may involve considerations not fully represented by conventional user-centered acceptance constructs. Multisite longitudinal studies using validated measures and real-world SAR exposure are needed to examine actual uptake and sustained use.

## Introduction

Over the past decade, socially assistive robots (SARs) have established a robust empirical foundation in pediatric hospital care, demonstrating significant efficacy in mitigating pain, anxiety, and negative affect among hospitalized children undergoing invasive or clinical procedures [1]. Paradoxically, this strong clinical evidence base has not translated into widespread institutional adoption, as routine deployment remains strictly limited to isolated pilot studies [2,3]. Recent implementation literature indicates that this research-to-practice gap is primarily organizational and psychological rather than technological, driven heavily by health care professionals’ (HCPs) anxieties regarding technological reliability, role clarity, and professional identity [4]. Consequently, while existing research extensively addresses whether SARs benefit patients, how HCPs conceptualize these technologies and what psychological or structural mechanisms drive their willingness to integrate them into daily clinical workflows remain poorly understood.

This implementation bottleneck stems from a foundational conceptual uncertainty regarding the unresolved conceptual and clinical status of SARs within clinical wards, where HCPs remain conflicted over whether these technologies function as tools, colleagues, toys, or entirely novel entities [4,5]. Within pediatric settings, this role ambiguity introduces distinct ethical and professional dilemmas, forcing providers to question whether delegating emotional-relational presence to a machine compromises professional identity or clinical appropriateness [2]. Crucially, empirical literature has not systematically evaluated the competing predictions generated by this conceptual ambiguity, leaving a critical theoretical tension unresolved between traditional operability-centric frameworks, which focus on ease of use and performance gains, and the relational-mediation hypothesis, which posits that a robot’s patient-facing emotional value is the primary driver of HCP acceptance [6,7].

Existing technology acceptance frameworks, including the Technology Acceptance Model (TAM) and the Unified Theory of Acceptance and Use of Technology (UTAUT), have primarily emphasized individual perceptions of usefulness, ease of use, and social or facilitating influences on technology adoption [8,9]. Although these frameworks provide a useful foundation for examining HCP acceptance, socially assistive technologies in pediatric care introduce additional considerations because they interact directly with children and are embedded within complex clinical and organizational environments. HCPs may therefore evaluate SARs not only according to their functional and operational characteristics but also in relation to child-robot interactions, trust, role appropriateness, and conditions for safe clinical implementation [2,10,11]. Trust is particularly relevant to autonomous and socially interactive technologies but is not explicitly represented as a distinct construct in UTAUT2. Accordingly, this study incorporated trust as an additional construct alongside the established UTAUT2 dimensions.

To address these gaps, this study used a convergent parallel mixed methods design to examine pediatric HCPs’ acceptance of SARs. The quantitative strand examined the associations of performance expectancy (PE), effort expectancy (EE), social influence (SI), facilitating conditions (FC), hedonic motivation (HM), and trust with behavioral intention (BI), while the qualitative strand explored HCPs’ experiential and contextual perspectives on SAR use in pediatric clinical practice. The quantitative and qualitative findings were integrated to identify areas of convergence, complementarity, and divergence and to examine perspectives not fully captured by the quantitative acceptance model. The study aimed to generate evidence to inform both the conceptualization of SAR acceptance and future implementation strategies in pediatric care.

## Methods

### Study Design

A convergent parallel mixed methods design [12] was used. Quantitative and qualitative data were collected concurrently between June and December 2025 and analyzed independently. The findings from both components were subsequently integrated using a joint display to examine convergence, complementarity, and divergence between HCPs’ technology acceptance and their experiential and contextual perspectives on SAR implementation.

### Ethical Considerations

Ethical approval was obtained from the Institutional Review Board (IRB) of Chang Gung Medical Foundation (IRB number 202500229B0; approved February 24, 2025) before data collection. All participants provided written informed consent before participation.

### Setting and Participants

The study was conducted at a tertiary medical center in northern Taiwan. Eligible participants were licensed physicians or registered nurses working in pediatric clinical settings and able to communicate in Mandarin Chinese. Recruitment and SAR exposure differed between the quantitative and qualitative strands.

### Quantitative Strand

#### Participants and Data Collection

Participants for the quantitative strand were recruited from HCPs working across the pediatric medical building, without restriction to specific pediatric units. Before completing the questionnaire, all participants viewed a standardized prerecorded video showing an SAR (Zenbo; ASUS Inc) interacting with a child in a pediatric care context. The video provided a common reference point for evaluating the SAR regardless of participants’ previous exposure to the technology. Accordingly, questionnaire responses reflected evaluations of a hypothetical use scenario rather than experience with routine clinical use of the SAR.

Prior clinical exposure to the SAR was assessed separately from exposure to the standardized video. During the same study period, a pediatric SAR intervention study was being conducted in selected clinical units [13]; therefore, some HCPs had previously observed the physical SAR interacting with children in the clinical setting or had clinical experience with the robot. Participants were categorized according to the reported frequency of prior clinical exposure as none, once, or ≥2 times.

#### Instrument Development

The questionnaire was developed through 4 stages: (1) pilot interviews with 5 clinical experts, a senior nurse with experience in robotics research (ASYL), a nurse manager experienced in technology acceptance questionnaire development (YWL), a senior pediatric nurse practitioner (MLC), a senior pediatric nurse with expertise in AI research (SMC), and a senior pediatric nurse specializing in child-centered pediatric care (SHC), to identify clinically relevant issues; (2) item development based on the UTAUT2 framework [9] and adaptation of items from 3 previous SAR studies [14-16]; (3) content validation by a 5-member expert panel (ASYL, YWL, MLC, SMC, and SHC; scale-level content validity index (average)=0.97; scale-level content validity index (universal agreement)=0.88); and (4) face-validity testing with 5 HCPs. Items were rated on a 5-point Likert scale from 1 (strongly disagree) to 5 (strongly agree).

The original 24-item instrument assessed 7 constructs: PE, EE, SI, FC, HM, trust, and BI. Following data collection, item-level evaluation identified 3 negatively worded items (Q4, Q7, and Q13) with corrected item-total correlations below the prespecified 0.20 criterion, despite appropriate reverse scoring. These items were therefore excluded based on their poor item-level performance. Although all 3 items were negatively worded, negative wording alone is unlikely to fully explain their poor performance because 2 other negatively worded items (Q16 and Q21) exceeded the retention criterion after reverse scoring. The specific reason for the low item-total correlations of Q4, Q7, and Q13 could not be determined from the available data. Their content, which concerned malfunction consequences, operational difficulty, and environmental unsuitability, may have been less applicable or interpreted differently by participants with limited direct clinical exposure to SARs; however, this remains a possible explanation rather than an empirically established cause. The resulting 21-item instrument was used for subsequent analyses. Full item wording, item-level statistics, and retention decisions are provided in Table S1 in the Multimedia Appendix 1.

#### Quantitative Data Analysis

Quantitative analyses were conducted using R (version 4.6.1; R Foundation for Statistical Computing). Negatively worded items retained in the final instrument were reverse coded before construct scores were calculated. Item-level psychometric evaluation used corrected item-total correlations, with 0.20 specified as the criterion for item retention. Following item refinement, internal consistency was assessed using Cronbach α and McDonald ω. For the 2-item FC subscale, the Spearman-Brown coefficient and interitem correlation were reported, consistent with recommendations for estimating reliability in 2-item scales [17].

Construct-specific single-factor models were estimated to obtain standardized factor loadings, composite reliability, and average variance extracted (AVE). A full simultaneous measurement-model confirmatory factor analysis was not estimated because of the sample size relative to model complexity. Discriminant validity was assessed using the heterotrait-monotrait ratio of correlations (HTMT), with 0.85 specified as the criterion [18]. Harman single-factor analysis assessed potential common method variance, and parallel analysis was conducted as an exploratory dimensionality check [19].

Participant characteristics and study constructs were summarized using frequencies, percentages, means and SDs, and Pearson correlations were used to examine bivariate associations. The primary inferential analysis was a standard multiple linear regression with simultaneous entry of PE, EE, SI, FC, HM, and TR to examine their associations with BI. Regression diagnostics included variance inflation factors, tolerance values, condition indices, residual diagnostics, the Durbin-Watson statistic, the Breusch-Pagan test, and Cook distance. Bias-corrected bootstrap 95% CIs were estimated for the standardized regression coefficients.

Secondary and sensitivity analyses examined the robustness of the primary findings. Incremental variance associated with HM and FC was evaluated using hierarchical regression, and sensitivity models were estimated after separately omitting FC, HM, and SI. Exploratory mediation examined the indirect association between EE and BI through trust in a 3-variable model that included only EE, trust, and BI and was not adjusted for the remaining predictors; standardized coefficients are reported. Exploratory moderation examined whether the trust-BI association differed between pediatric intensive care unit (PICU) and non-PICU participants. These analyses were not prespecified and were interpreted as exploratory.

Group differences by professional role and clinical unit were examined using independent-samples *t* tests and one-way ANOVA with Bonferroni-adjusted post hoc comparisons, respectively. Because the clinical unit subgroups were unequal in size, particularly pediatric surgery (n=11), a sensitivity analysis estimated the minimum detectable effect size for each pairwise unit contrast at a Bonferroni-adjusted α of 0.0083 and 80% power, and the pairwise unit contrasts were interpreted as exploratory. Prior clinical SAR exposure was examined separately in an exploratory descriptive analysis that was not powered for subgroup contrasts. Given the small and unequal exposure groups, differences in BI and overall acceptance across the 3 exposure groups were evaluated primarily using the Kruskal-Wallis test, followed by Mann-Whitney *U* tests for pairwise comparisons with a Bonferroni-adjusted significance threshold (α=.017). Effect sizes were reported for pairwise comparisons. Parametric one-way ANOVA, Welch tests, and bootstrap 95% CIs were retained as supplementary robustness analyses and are reported in the Multimedia Appendix 1. Overall acceptance was defined as the mean of PE, EE, SI, FC, HM, and TR, excluding BI.

An a priori power analysis using G*Power 3.1 (Heinrich Heine University Düsseldorf) [20], assuming Cohen *f*²=0.15, α=.05, and 6 predictors, determined that 98 participants were required for 80% power [21]; the achieved sample of 125 therefore exceeded this requirement for the primary multiple regression analysis. This power calculation did not apply to the exploratory prior-exposure subgroup comparisons. A sensitivity analysis subsequently estimated the minimum detectable effect size for pairwise comparisons at α=.017 and 80% power based on the observed subgroup sizes.

### Qualitative Strand

Participants in the qualitative strand were purposively recruited primarily from the PICU and pediatric surgery, the 2 units participating in the hospital’s SAR pilot during the study period. Eligibility for the qualitative strand required prior clinical exposure to the physical SAR, defined as having observed the robot interacting with children in the clinical setting or having prior clinical experience with the robot. This differed from the quantitative strand, in which prior clinical SAR exposure was not required and all participants viewed a standardized prerecorded video before completing the questionnaire. Purposive sampling was used to achieve variation in professional role and clinical seniority and to capture diverse perspectives on the clinical use and implementation of SARs.

Participants who took part in the qualitative strand were interviewed before completing the quantitative questionnaire to minimize potential priming from the structured UTAUT2 items. Data collection and preliminary analysis proceeded iteratively, with recruitment continuing until additional interviews no longer contributed substantively new information relevant to the study aims. Because participation in each strand was voluntary and independent, not all interview participants subsequently completed the questionnaire; the 2 samples therefore overlapped only partially. The final qualitative sample comprised 17 HCPs from the nursing and medical professions.

The semistructured interview guide (Table S2 in Multimedia Appendix 1) covered 8 domains related to participants’ perceptions and anticipated use of SARs in pediatric clinical practice while allowing participants to elaborate on issues they considered important. Interviews lasted approximately 30‐60 minutes and were audio-recorded with written informed consent. Interviews were conducted in Mandarin Chinese and transcribed verbatim using automated transcription software. All transcripts were subsequently checked against the original audio recordings for accuracy. Quotations presented in this paper were translated into English by the research team.

### Qualitative Data Analysis

Interview data were analyzed using thematic analysis informed by Braun and Clarke [22]. Analysis was iterative and involved familiarization with the transcripts, generation of initial codes, identification of patterns across codes, development and review of candidate themes, and refinement and definition of the final themes. Although the interview guide (Multimedia Appendix 1) addressed concepts relevant to technology acceptance, coding was primarily inductive and remained open to perspectives not anticipated by the UTAUT2 framework.

Two researchers (YHL and FYH) independently coded all verified transcripts and compared codes and interpretations during regular analytic meetings. Disagreements were resolved through discussion, with the corresponding author reviewing relevant excerpts when needed. Candidate themes were repeatedly compared with the complete dataset and refined to ensure that they represent coherent patterns across participants rather than isolated responses. Coding was conducted manually without dedicated qualitative analysis software. Consistent with the interpretive orientation of thematic analysis, themes were developed based on their relevance to the research aims and the patterning of meaning across the dataset rather than solely on the frequency with which particular issues were mentioned. Participant counts were therefore not used as a criterion for determining thematic importance. A condensed coding framework showing the relationships among initial codes, categories, and final themes is provided in Table S9 in the Multimedia Appendix 1.

Trustworthiness was addressed according to Lincoln and Guba [23] criteria of credibility, transferability, dependability, and confirmability. Credibility was supported through purposive sampling and iterative engagement with the interview data. Dependability and confirmability were strengthened through independent coding, analytic discussions, repeated comparison of candidate themes with the original transcripts, and documentation of coding decisions. Five transcripts were re-examined approximately 1 month after initial coding to assess the stability of coding interpretations. Transferability was supported by describing participant characteristics and the clinical contexts represented in the qualitative sample.

### Mixed Methods Integration

Quantitative and qualitative findings were integrated after the independent analyses of both strands [12]. A joint display was developed to compare quantitative constructs and statistical findings with corresponding qualitative themes. Findings were examined for convergence, in which both strands supported similar interpretations; complementarity, in which qualitative findings elaborated on or contextualized quantitative results; and divergence, in which findings differed across the 2 strands. Integrated interpretations were then developed to identify areas in which experiential and contextual findings extended or challenged relationships observed in the quantitative model.

Because the qualitative strand purposively sampled HCPs with direct clinical exposure to the physical SAR from the 2 pilot units, whereas the quantitative strand included a broader pediatric workforce, most of whom evaluated the SAR primarily based on a standardized video, the 2 strands represented partially overlapping but nonequivalent samples with different levels of clinical SAR exposure. Integration was therefore used to juxtapose findings across these complementary perspectives rather than to assume direct equivalence between the 2 strands. Differences in clinical setting, sampling, and prior SAR exposure were explicitly considered when interpreting areas of convergence, complementarity, and divergence.

## Results

### Participant Characteristics

A total of 125 HCPs completed the quantitative survey, and 17 HCPs participated in qualitative interviews (Table 1). The quantitative sample comprised 100 (80%) nurses and 25 (20%) physicians from pediatric internal medicine, the PICU, pediatric emergency care, and pediatric surgery. Most participants (104/125, 83.2%) had no prior clinical SAR experience, whereas 11 of 125 (8.8%) reported 1 prior exposure and 10 of 125 (8%) reported 2 or more exposures.

**Table 1.** Participant characteristics of the quantitative and qualitative samples.

Variables	Quantitative (N=125), n (%)	Qualitative (n=17^a^), n (%)
Sex		
Female	105 (84.0)	12 (70.6)
Male	20 (16.0)	5 (29.4)
Age (years)		
20‐30	37 (29.6)	6 (35.3)
31‐40	44 (35.2)	3 (17.6)
41‐50	33 (26.4)	4 (23.5)
51‐60	8 (6.4)	4 (23.5)
>60	3 (2.4)	0 (0)
Education level		
Bachelor’s degree	96 (76.8)	14 (82.4)
Master’s degree	22 (17.6)	3 (17.6)
Associate and other degree	7 (5.6)	0 (0)
Profession		
Nursing (total)	100 (80.0)	10 (58.8)
Clinical nurse	89 (71.2)	8 (47.1)
Head nurse	7 (5.6)	2 (11.8)
Nurse practitioner	3 (2.4)	0 (0)
Supervisor	1 (0.8)	0 (0)
Physician (total)	25 (20.0)	7 (41.2)
Attending physician (VS)	8 (6.4)	4 (23.5)
Resident and Chief resident	17 (13.6)	3 (17.6)
Clinical unit		
Pediatric internal medicine	44 (35.2)	0 (0)
PICU^b^	39 (31.2)	4 (23.5)
Pediatric emergency	31 (24.8)	0 (0)
Pediatric surgery	11 (8.8)	13 (76.5)
Prior clinical SAR exposure^c^		
None	104 (83.2)	0 (0)
Once	11 (8.8)	14 (82.4)
Twice or more	10 (8.0)	3 (17.6)
Clinical experience (years)		
Mean (SD)	—^d^	14.5 (11.1)
Range	—	1‐30

a The quantitative and qualitative samples overlapped only partially because interviews preceded questionnaire completion, and participation in each strand was voluntary; the columns therefore represent partially overlapping, nonnested samples and are not additive.

b PICU: pediatric intensive care unit.

c Prior clinical SAR (socially assistive robot) exposure refers to previous exposure to the physical SAR in the clinical setting, including observing the SAR interacting with children or having prior clinical experience with the robot; exposure to the standardized prerecorded video was not included.

d Not systematically recorded for that strand.

The qualitative sample comprised 10 nurses of 17 participants (58.8%) and 7 physicians of 17 participants (41.2%), with a mean clinical experience of 14.5 (SD 11.1) years. Participants were drawn from pediatric surgery (13/17, 76.5%) and the PICU (4/17, 23.5%).

### Quantitative Results

The final 21-item instrument demonstrated high overall internal consistency (Cronbach α=0.942; McDonald *ω*=0.951). Across the multi-item constructs, Cronbach α ranged from 0.651 for trust to 0.913 for BI, whereas McDonald ω ranged from 0.708 for trust to 0.945 for BI. For the 2-item FC construct, the Spearman-Brown coefficient was 0.662 (interitem *r*=0.495). Construct-specific single-factor analyses showed composite reliability ranging from 0.708 for trust to 0.916 for BI and AVE ranging from 0.476 to 0.785; TR was the only construct with an AVE below 0.50 (AVE=0.476). HTMT values exceeded the prespecified 0.85 criterion for 7 construct pairs (EE-FC=0.969, SI-FC=0.950, SI-HM=0.936, trust-HM=0.914, BI-HM=0.906, FC-HM=0.882, and SI-BI=0.862), indicating limited discriminant validity among these constructs; these constructs therefore could not be assumed to represent empirically distinct dimensions in this sample. The retained negatively worded items Q16 and Q21 had comparatively low standardized factor loadings (λ=0.227 and 0.333, respectively). Construct-level reliability and descriptive statistics are presented in Table 2; complete standardized factor loadings, composite reliability, and AVE are provided in Table S3, HTMT results are provided in Table S4, and item-level statistics are provided in Table S1 in Multimedia Appendix 1.

**Table 2.** Scale reliability and construct means (N=125).

Construct	Items (n)	Cronbach α	McDonald ω	Mean^a^ (SD)
Performance expectancy	3	0.877	0.878	4.13 (0.65)
Effort expectancy	3	0.782	0.788	3.78 (0.68)
Social influence	3	0.801	0.802	4.04 (0.58)
Facilitating conditions	2	0.662^b^	—^c^	3.75 (0.72)
Hedonic motivation	4	0.735	0.764	3.73 (0.63)
Trust	3	0.651	0.708	3.35 (0.66)
Behavioral intention	3	0.913	0.945	4.04 (0.66)
Full 21-item scale^d^	21	0.942	0.951	3.83 (0.54)

a Mean score on 5-point Likert scale.

b For the 2-item facilitating conditions construct, reliability was assessed using the Spearman-Brown coefficient (0.662; interitem *r*=.495); McDonald ω is not calculated for 2-item constructs due to model under-identification.

c Not applicable.

d Full 21-item scale refers to the retained items after refinement; this row includes the behavioral intention items and is therefore not equivalent to overall acceptance, which was defined as the mean of the 6 predictor constructs excluding behavioral intention.

Harman single-factor analysis showed that the first unrotated factor accounted for 51.7% of the total variance, exceeding the commonly used 50% threshold and raising concern that common method variance may have contributed to the observed associations. Exploratory parallel analysis showed a dominant first factor and did not support retention of the hypothesized 7-factor structure in this sample (Figure S1 in Multimedia Appendix 1). These findings were treated as measurement limitations rather than used to redefine the theory-derived UTAUT2 constructs, and construct-specific associations were interpreted accordingly. PE had the highest mean score (mean 4.13, SD 0.65), followed by SI (mean 4.04, SD 0.58) and BI (mean 4.04, SD 0.66), whereas trust had the lowest mean score (mean 3.35, SD 0.66). Overall acceptance was 3.83 (SD 0.54). Construct-level descriptive statistics are presented in Table 2, and Pearson correlations are provided in Table S5 in Multimedia Appendix 1.

### Factors Associated With BI

The multiple regression model was statistically significant and accounted for 72.7% of the observed variance in BI (*R*²=.727, adjusted *R*²=.714; *F*_6,118_=52.50; *P*<.001). SI had the largest standardized coefficient (β=0.348; *P*<.001), followed by HM (β=0.307; *P*<.001), PE (β=0.238; *P*=.001), and TR (β=0.134; *P*=.047). EE (β=0.075; *P*=.30) and FC (β=−0.125; *P*=.12) were not significantly associated with BI in the adjusted model. Because several predictors showed limited discriminant validity, these coefficients were interpreted as associations conditional on the other correlated predictors rather than as evidence of distinct construct effects. Complete regression coefficients, SEs, 95% CIs, and variance inflation factors (VIFs) are presented in Table 3.

**Table 3.** Multiple regression predicting behavioral intention (N=125).

Predictor	B^a^ (95% CI)	SE	β^b^	*t* test (*df*)	*P* value	VIF^c^
Performance expectancy	0.239 (0.094 to 0.384)	0.074	0.238	3.26 (118)	.001	2.31
Effort expectancy	0.073 (−0.065 to 0.211)	0.070	0.075	1.05 (118)	.30	2.23
Social influence	0.391 (0.201 to 0.581)	0.096	0.348	4.07 (118)	<.001	3.16
Facilitating conditions	−0.115 (−0.258 to 0.029)	0.073	−0.125	−1.58 (118)	.12	2.70
Hedonic motivation	0.318 (0.161 to 0.475)	0.079	0.307	4.01 (118)	<.001	2.54
Trust	0.133 (0.002 to 0.265)	0.066	0.134	2.01 (118)	.047	1.93

a B: unstandardized regression coefficient.

b β: standardized coefficient

c VIF: variance inflation factor.

### Regression Diagnostics

Regression diagnostics did not indicate substantial multicollinearity, influential observations, or major violations of model assumptions. Although the maximum condition index was 33.3, the variance-decomposition pattern did not indicate substantial multicollinearity affecting the regression estimates.

### Secondary and Sensitivity Analyses

Bias-corrected bootstrap 95% CIs for the standardized regression coefficients excluded zero for SI (0.129-0.574), HM (0.116-0.474), PE (0.019-0.439), and trust (0.011-0.257), but included zero for EE (−0.087 to 0.242) and FC (−0.294 to 0.048), broadly supporting the primary model. In hierarchical analyses, HM explained an additional 3.3% of the variance in BI (Δ*R*²=0.033, *F*_1,118_=14.09; *P*<.001), whereas FC did not contribute significant incremental variance (Δ*R*²=0.006, *F*_1,118_=2.50; *P*=.12). Sensitivity models separately excluding FC, HM, or SI left the coefficients for SI, HM, and PE substantively unchanged, whereas the association between trust and BI was no longer statistically significant when FC was excluded (β=0.119; *P*=.075), indicating that the trust association was sensitive to model specification. These sensitivity analyses did not address the construct overlap identified by the HTMT analysis.

In additional post hoc exploratory analyses, the indirect association between EE and BI through trust was statistically significant (standardized indirect effect=0.245, 95% CI 0.149-0.382; 5000 bias-corrected bootstrap resamples), whereas the trust×PICU interaction was not statistically significant (β=−0.115, Δ*R*²=.002; *P*=.50). Given the post hoc nature of these analyses and the limited convergent and discriminant validity of TR, these findings were considered hypothesis-generating and were not interpreted as evidence of causal mediation or moderation.

### Exploratory Analysis of Prior Clinical SAR Exposure

Given the small and unequal exposure groups (n=104, 11, and 10), this analysis was considered descriptive and exploratory. Mean BI scores were 3.94 (SD 0.62), 4.30 (SD 0.64), and 4.77 (SD 0.52) among participants with no prior clinical exposure, 1 previous exposure, and ≥2 previous exposures, respectively. Kruskal-Wallis tests indicated differences across exposure groups for BI and overall acceptance (both *P*<.001). Bonferroni-adjusted pairwise comparisons showed differences between the no-exposure and ≥2-exposure groups for both outcomes and between the no-exposure and 1-exposure groups for overall acceptance. However, sensitivity analysis indicated that the small and unequal subgroups were powered to detect only very large pairwise differences. These findings were therefore considered descriptive and hypothesis-generating. Complete effect sizes, pairwise comparisons, sensitivity analyses, and parametric robustness analyses are provided in Table S6 in Multimedia Appendix 1.

### Profession and Clinical Unit Comparisons

No statistically significant differences were observed between physicians and nurses in any UTAUT2 construct, BI, or overall acceptance (all *P*≥.19; Table S7 in Multimedia Appendix 1).

Scores differed across clinical units for trust (*F*_3,121_=7.97; *P*<.001, η²=0.165), HM (η²=0.119), overall acceptance (η²=0.105), FC (η²=0.087), BI (η²=0.084), and SI (η²=0.068), with generally lower scores in the PICU and higher scores in pediatric surgery (Table S8 in Multimedia Appendix 1).

Bonferroni-adjusted post hoc comparisons indicated that PICU participants had lower trust, HM, and overall acceptance scores than participants from pediatric surgery (adjusted *P*<.001, *P*=.002, and *P*=.003, respectively). PICU participants also had lower trust than participants from pediatric internal medicine (adjusted *P*=.02), whereas the corresponding contrasts for HM and overall acceptance did not meet the adjusted threshold (adjusted *P*=.75 and *P*=.33, respectively). Exploratory PICU-versus-other-unit contrasts showed a similar pattern for trust, HM, BI, and overall acceptance; complete results are provided in Table S8 in Multimedia Appendix 1. The unit subgroups were also unequal in size, and sensitivity analysis indicated that the pairwise unit contrasts could detect only large standardized differences (minimum detectable Cohen *d*=0.78‐1.28, with the largest values for contrasts involving pediatric surgery, n=11). These pairwise unit comparisons were therefore also interpreted as exploratory and hypothesis-generating.

### Qualitative Results

Thematic analysis of the 17 interviews generated 6 themes and 31 categories reflecting HCPs’ perceptions of SAR adoption in pediatric care (Table S9 in Multimedia Appendix 1). The themes encompassed (1) emotional and stress-related challenges of hospitalized children, (2) functional roles and clinical positioning of SARs, (3) health care providers’ attitudes toward SARs, (4) child-centered design considerations, (5) practical considerations for clinical implementation, and (6) institutional decision-making and management. The thematic structure and representative quotations are presented in Table 4. A detailed coding framework showing the categories within each theme is provided in Table S9 in Multimedia Appendix 1.

**Table 4.** Themes and representative quotations (N=17).

Theme	Representative quotation^a^
Theme 1. Emotional and stress-related challenges of hospitalized children	“What stands out most is how frightened they are of strangers. To a child, a group of people in white coats turning up at the bedside to do things to them might as well be ghosts.” (HCP^b^ 16, PICU^c^)
Theme 2. Functional roles and clinical positioning of SARs^d^	“Children warm to a robot faster than they warm to us. It meets them as an equal, so they will open to it in a way they never would with a doctor or a nurse standing over them.” (HCP 13, pediatric surgery)
Theme 3. Health care providers’ attitudes toward SARs	“What separates us from a machine is that we are human. A machine is a dead thing. Whatever it says was written somewhere else; it does not come out of a real exchange between two people.” (HCP 16, PICU)
Theme 4. Child-centered design considerations	“It must run itself and set off when it is due. If a nurse must switch it on every single time, the whole point of it is gone.” (HCP 11, pediatric surgery)
Theme 5. Practical considerations for clinical implementation	“If we cannot vouch for the source, do not let it search the internet. Turn it loose and it will hand out something wrong, and where medical information is concerned that worries us.” (HCP 13, pediatric surgery)
Theme 6. Institutional decision-making and management	“Say the robot puts in a central line and nicks the lung. Who carries that? It is the same question as when I send a resident to do a procedure and the resident gets it wrong, and we do not have an answer to that one either.” (HCP 16, PICU)

a Quotations were translated from the original Mandarin Chinese by the authors.

b HCP: health care professional.

c PICU: pediatric intensive care unit.

d SAR: socially assistive robot.

Across the interviews, participants described SARs as potentially valuable not only for task-related support but also for emotional engagement, distraction, companionship, and communication with children. Perceived usefulness was therefore closely linked to the robot’s capacity to establish developmentally appropriate and emotionally responsive interactions.

At the same time, participants emphasized practical and clinical concerns, including ease of operation, device reliability, infection control, privacy, workflow compatibility, and responsibility when technical problems occurred. Trust-related concerns were articulated in distinctive terms by PICU participants, who described greater uncertainty regarding the use of SARs in clinically complex and safety-sensitive environments.

Participants also described adoption as an organizational rather than solely individual decision. Leadership support, institutional policies, staff readiness, resource availability, and clarity regarding accountability were viewed as important conditions for routine implementation. Whereas practical usability and operational burden were elaborated in detail across accounts, peer or supervisory expectations were not articulated by participants as a consideration bearing on their willingness to use SARs.

### Mixed Methods Integration

Integration identified areas of convergence, complementarity, and divergence between the quantitative and qualitative findings (Table S10 in Multimedia Appendix 1). PE showed convergence across strands, with its positive association with BI corresponding to qualitative descriptions of the functional value of SARs in pediatric care.

Complementarity was observed for trust, with qualitative accounts describing concerns regarding reliability, information accuracy, privacy, accountability, and other implementation conditions. For HM, the quantitative construct reflected HCPs’ own enjoyment, whereas the qualitative accounts focused primarily on children’s enjoyment, comfort, and engagement; this pattern was therefore classified as conceptual divergence.

Divergence was also observed for SI and EE. SI had the largest standardized coefficient in the quantitative analysis, whereas normative expectations from peers or supervisors were not articulated as a consideration in the qualitative accounts. In contrast, ease of use and operational burden were elaborated in detail in the qualitative accounts, whereas EE was not significantly associated with BI in the adjusted quantitative model. Exploratory findings related to prior clinical SAR exposure are also summarized in the joint display.

## Discussion

### Principal Findings

This mixed methods study found that SI, HM, and PE had the largest standardized coefficients in the model of HCPs’ intention to adopt SARs, whereas the association for trust was small and sensitive to model specification. Because several of these constructs showed limited discriminant validity and common method variance could not be excluded, these associations should be regarded as provisional rather than as evidence of distinct determinants of intention. The qualitative findings provided a complementary perspective, showing that HCPs evaluated SARs in terms of their functional contribution to care, their capacity to engage and support children, and the institutional conditions relevant to implementation.

Importantly, the child-centered relational value identified qualitatively was conceptually distinct from UTAUT2-defined HM, which reflects the technology user’s own enjoyment. This distinction is particularly relevant in pediatric care, where the perceived value of SARs may derive from their capacity to engage, distract, comfort, and interact with children rather than from HCPs’ own enjoyment of using the technology. Previous pediatric SAR research has similarly emphasized children’s engagement, emotional support, and interactive experiences as important features of robot-assisted care [1,7,24]. Accordingly, the qualitative findings concerning children’s enjoyment and engagement should not be interpreted as explaining or validating the quantitative association between HM and BI.

Trust-related concerns emerged in the qualitative findings, particularly in relation to reliability, information accuracy, privacy, and accountability, and were articulated in distinctive terms by PICU participants. Previous research has similarly shown that trust in SARs can vary with robot performance, prior experience, user characteristics, and interaction context [25], while technological reliability, ethical safeguards, training, role clarity, and organizational readiness have been identified as relevant considerations for SAR implementation [4,26]. In this study, however, the quantitative association between trust and BI was small and sensitive to model specification, and TR showed limited convergent and discriminant validity. Trust should therefore be considered a contextual concern identified in the qualitative data rather than a robust independent quantitative determinant of BI. Taken together, the integrated findings highlight 3 considerations in HCPs’ evaluations of pediatric SARs: clinical utility, child-centered relational value, and conditions relevant to safe implementation.

### SI and BI

SI had the largest standardized coefficient in the present regression model. This finding is consistent with broader UTAUT2 research identifying SI as an important correlate of BI [27] and with evidence that health technology adoption is associated with social and contextual factors beyond individual evaluations of the technology [28]. The observed association suggests that HCPs’ intentions to adopt emerging technologies may be related to the professional and organizational environments in which adoption is considered.

Notably, the prominence of SI in the quantitative model was not mirrored in the qualitative findings. Peer or supervisory expectations were not articulated by participants as bearing on their willingness to use SARs; instead, accounts were organized around child engagement, technological reliability, workflow compatibility, and accountability. This divergence may partly reflect differences in how the 2 methods elicited perceptions. However, it also raises the possibility that normative SI was not a particularly salient consideration for clinically exposed HCPs.

The psychological meaning of the quantitative SI association therefore remains uncertain. No cognitive interviewing was conducted to determine how participants interpreted the SI items, which may have captured normative influence as intended or a more general favorable evaluation of SARs. Limited discriminant validity, the dominant factor identified by parallel analysis, and the potential contribution of common method variance further limit construct-specific interpretation. Differences in sampling and prior SAR exposure between the qualitative and quantitative strands may also have contributed to the observed divergence. Accordingly, SI should be interpreted as a statistical correlate of BI rather than as evidence of a clearly defined normative mechanism. Future research using cognitive interviewing and more differentiated measures of interpersonal influence, leadership endorsement, organizational norms, and institutional support may help clarify this relationship.

### HM and Child-Centered Relational Value

HM had the second largest standardized coefficient in the regression model. The qualitative findings, however, did not emphasize HCPs’ own enjoyment; rather, participants described the value of SARs in terms of their capacity to engage children, provide distraction and companionship, and create interactions perceived as less threatening than conventional clinical encounters. Child-centered design features, including age-appropriate appearance, eye-level interaction, and autonomous engagement, were also considered important. These findings are consistent with pediatric SAR research emphasizing engagement, emotional support, adaptive distraction, and developmentally appropriate interaction as important features of robot-assisted care [1,2,7,29].

Within UTAUT2, HM refers to the enjoyment or pleasure experienced by the technology user—in this study, the HCPs—and the HM items were adapted to assess respondents’ own enjoyment. In contrast, interview participants primarily described children’s enjoyment, comfort, engagement, and willingness to interact with the SAR. The 2 strands therefore appear to capture conceptually different phenomena, and the qualitative findings should not be used to explain or validate the quantitative HM association. We refer to the qualitative dimension as child-centered relational value, reflecting the value HCPs attribute to a SAR’s capacity to engage, comfort, and interact with children.

The meaning of the quantitative HM association nevertheless remains uncertain. Without cognitive interviewing, it is not possible to determine how HCPs interpreted the adapted HM items in this pediatric, patient-facing context. In addition, limited discriminant validity and potential common method variance raise the possibility that HM partly reflected a broader favorable evaluation of SARs rather than a clearly distinct hedonic construct. Future research should separately assess HCP-centered HM and child-centered relational value, use cognitive interviewing to examine how HCPs interpret HM items, and evaluate whether child-centered relational value represents a distinct dimension relevant to technology acceptance in pediatric care.

### EE and Implementation Considerations

Although EE was not significantly associated with BI in the adjusted model, usability and operational considerations were elaborated in detail in the qualitative accounts. Participants discussed not only ease of use but also workflow compatibility, training, organizational preparation, and integration into clinical practice, consistent with previous SAR implementation research [26]. These findings suggest that individual perceptions of ease of use and broader requirements for clinical implementation may represent related but distinct considerations.

Interpretation of the EE finding also requires caution because the negatively worded item concerning operational difficulty was removed during item refinement. Although its removal improved internal consistency, it may have narrowed the content represented by the retained EE items, particularly regarding operational burden. The qualitative participants’ direct clinical SAR exposure may also have made practical implementation concerns more salient than they were for survey respondents who primarily viewed the standardized video. Accordingly, the nonsignificant quantitative association for EE should not be interpreted as evidence that usability or operational considerations are unimportant in evaluating pediatric SARs.

### Trust and Context-Specific Implementation Concerns

Trust-related concerns were evident in HCPs’ evaluations of SARs, with PICU accounts specifically emphasizing the consequences of technological failure in high-acuity care. Qualitatively, participants emphasized technological reliability, information accuracy, privacy and data security, and accountability, while those in the PICU described a particularly low tolerance for technological failure in high-acuity care. Exploratory quantitative comparisons showed a similar pattern, with lower trust reported in the PICU than in other pediatric settings. Together, these findings suggest that trust-related evaluations may vary with clinical context and that high-acuity environments may place greater emphasis on assurance when the perceived consequences of technological failure are greater. This interpretation is consistent with research on clinical AI emphasizing calibrated trust and appropriate reliance rather than uniformly maximizing trust [30,31], as well as studies identifying reliability, accountability, and safeguards as important considerations in SAR and AI implementation [2,4,11]. However, because the unit comparisons were exploratory and this study assessed perceived trust rather than actual reliance behavior, this interpretation remains provisional.

The quantitative contribution of trust should nevertheless be interpreted cautiously. Trust showed limitations in convergent and discriminant validity, and its association with BI was small and sensitive to model specification. Moreover, the moderation analysis did not provide evidence that the trust-BI association differed between PICU participants and those from other units. The findings therefore provide stronger support for the contextual salience of trust-related concerns than for a distinct and robust quantitative association between trust and BI across the full sample.

The qualitative findings further suggest that trust-related evaluations of pediatric SARs may be multidimensional and context-dependent. Langer et al [25] similarly conceptualized trust in socially assistive robotics within the patient-clinician-robot relationship, emphasizing robot performance, prior experience, user characteristics, and interaction context. In this study, concerns regarding reliability, information credibility, privacy and security, and accountability may represent different aspects of trust-related evaluation that are not fully captured by a single general trust construct. Future research should therefore examine context-sensitive measures of trust across pediatric clinical settings.

### Clinical and Theoretical Implications

From a theoretical perspective, the findings suggest that HCPs’ evaluations of socially interactive technologies in pediatric care may involve considerations not fully represented by conventional technology acceptance constructs. PE was associated with BI, consistent with broader technology acceptance research [27,28], while the qualitative findings highlighted child engagement and relational interaction as additional considerations. Clinical utility and child-centered relational value may therefore represent conceptually complementary aspects of HCPs’ evaluations of pediatric SARs. However, the latter emerged from the qualitative data and was not directly measured as a quantitative construct in the present study. Together with social, trust-related, and implementation concerns identified across the 2 strands, these findings provide a basis for further examining how conventional technology acceptance frameworks may be adapted to socially interactive technologies in pediatric care.

From an implementation perspective, the qualitative findings point to considerations that may be relevant when SARs are evaluated for use within pediatric clinical systems. The Nonadoption, Abandonment, Scale-up, Spread, and Sustainability (NASSS) framework provides a useful perspective for considering interactions among the technology, adopters, organization, wider context, and adaptation over time [32]. In this study, participants raised issues related to children’s needs, clinical workflows, organizational resources, governance, technological reliability, privacy and ethical safeguards, and adaptation to specific care contexts. Similar considerations have been identified in previous SAR implementation research [4,26,29].

Because the quantitative strand assessed BI rather than actual adoption, and most survey participants evaluated the SAR primarily through a standardized video rather than sustained clinical use, these practical implications should be considered hypotheses for future implementation research rather than evidence-based implementation strategies. Future studies could examine whether presenting SAR functions within clinically relevant workflows, including opportunities to observe child-facing interactions, affects HCPs’ evaluations of their clinical and relational value. They could also examine how reliability, information accuracy, privacy and data security, maintenance, workflow integration, accountability, and risk-sensitive assurance requirements affect actual uptake and sustained use. The potential role of direct clinical exposure also warrants prospective evaluation, as the exploratory exposure-group findings in the present study were based on small, unequal, and underpowered subgroups and cannot establish causal or exposure-response relationships.

### Limitations

Several limitations should be considered when interpreting these findings. First, this was a single-site study conducted at a tertiary medical center in Taiwan. Institutional characteristics, staffing patterns, clinical workflows, and organizational norms may differ across health care systems, which may limit the generalizability of the findings. The nurse-to-patient ratio of approximately 1:10 on the participating general pediatric wards should also be considered when interpreting the findings across settings.

Second, the cross-sectional design precludes causal inference, and the study assessed BI rather than actual adoption or use. Most quantitative participants evaluated the SAR primarily based on a standardized video rather than sustained clinical use. Given the well-documented intention-behavior gap [33], the factors associated with BI in this study may not predict real-world SAR adoption, which is also shaped by organizational, technical, and resource conditions not directly examined here [32]. Moreover, the prior-exposure subgroups were small and unequal, and sensitivity analysis indicated that pairwise comparisons were powered to detect only very large effects. The pairwise comparisons across clinical units were similarly limited, because the unit subgroups were unequal in size and the smallest unit contributed 11 participants. Findings related to prior exposure and to differences between clinical units should therefore be considered exploratory and hypothesis-generating.

Third, several measurement limitations warrant consideration. Three negatively worded items from PE, EE, and FC were removed because of poor item-level performance. Although removal improved internal consistency, these items addressed malfunction consequences, operational difficulty, and environmental unsuitability—issues that were also raised in the qualitative interviews. Their removal may therefore have narrowed the content coverage of these constructs, particularly with respect to negatively framed implementation concerns that were also reflected in the qualitative interviews. FC was represented by only 2 items and showed modest reliability, while trust showed limited convergent validity. More broadly, limited discriminant validity and the parallel analysis raised concerns about the empirical distinctiveness of the theory-derived constructs in this sample. The regression coefficients should therefore not be interpreted as independent construct effects. Common method variance is also a concern because all quantitative constructs were assessed by self-report at a single time point. Procedural remedies recommended for reducing common method bias [34] were incorporated at the design stage, including anonymous administration, assurance that there were no right or wrong answers, separation of items belonging to different constructs within the questionnaire, and inclusion of reverse-worded items. Statistical remedies were more limited: no marker variable was administered, and the available sample did not support estimation of a measurement model incorporating a latent method factor, so the assessment was restricted to the Harman single-factor test. Evidence on the consequences of method variance indicates that, for linear main effects of the kind estimated here, variance shared between predictors and the criterion generally inflates rather than attenuates observed relationships [35]. Common method variance may therefore have inflated some of the observed associations, including the relatively large SI-BI and HM-BI associations. However, because no marker variable or latent method factor was available, neither the magnitude nor the construct-specific impact of common method variance can be determined from the current data. The standardized coefficients and their relative ordering should therefore be interpreted cautiously. Their true magnitudes may be smaller than the standardized coefficients suggest. Because the amount of shared method variance cannot be quantified without a marker variable, the extent of any such inflation cannot be estimated from the current data, and the relative ordering of the predictors should likewise be regarded as provisional. In addition, no cognitive interviewing was conducted to examine how participants interpreted the adapted items [36]. Collectively, these limitations warrant caution in construct-specific interpretation and support further validation of the instrument in larger pediatric HCP samples.

Fourth, the 2 strands involved partially overlapping but non-equivalent samples with different levels of clinical SAR exposure. Because participation in each strand was voluntary, interview participants were not required to complete the questionnaire, and the qualitative sample is therefore not a subset of the quantitative sample. The qualitative sample was concentrated in pediatric surgery and the PICU, the 2 units participating in the hospital’s SAR pilot, and all interview participants had direct clinical exposure to the physical SAR. In contrast, the quantitative sample represented a broader range of pediatric settings, and most respondents had no prior clinical SAR exposure. The qualitative findings may therefore more strongly reflect the perspectives of clinically exposed HCPs in surgical and intensive care contexts. Consequently, the integration cannot determine whether areas of complementarity or divergence, particularly for SI, HM, and EE, reflect differences in method, clinical setting, sampling, or prior SAR exposure. The integrated findings should therefore be interpreted as a juxtaposition of complementary but nonequivalent perspectives rather than as direct cross-strand confirmation.

Finally, several secondary analyses, including those involving prior clinical SAR exposure, mediation, and moderation, were not prespecified and should be considered hypothesis-generating. These findings require confirmation in prospectively designed studies with adequate sample sizes.

### Future Directions

Future research should extend these findings through larger, multisite studies across hospitals with different organizational structures, resources, and pediatric populations. Studies should include diverse clinical settings with differing levels of acuity, procedural distress, and workflow demands to examine whether HCP evaluations of SARs vary across care contexts. Longitudinal research from preimplementation through sustained clinical use is particularly needed to examine how perceptions evolve with direct SAR experience and whether BIs translate into actual adoption and use. Future mixed methods studies should also recruit qualitative and quantitative participants from comparable clinical settings and levels of SAR exposure to strengthen cross-strand integration.

Future implementation research should also evaluate the technical and clinical readiness of SARs, including reliability, operational stability, information accuracy, privacy and data security, workflow integration, and adaptability across clinical contexts. Measurement development is another priority. SAR acceptance measures should undergo cognitive pretesting and independent validation in larger and more diverse pediatric HCP samples, with particular attention to construct distinctiveness and common method bias. Future studies should distinguish HCP-centered HM from perceived child engagement and examine whether child-centered relational value represents a distinct and measurable dimension of HCP evaluation. Context-sensitive measures of trust should also differentiate concerns such as reliability, information credibility, privacy, accountability, and appropriate reliance. Longitudinal mixed methods research could then examine how technological, relational, and organizational factors are associated with actual uptake and sustained use over time.

### Conclusions

Pediatric HCPs’ intentions to adopt SARs were associated with SI, HM, and PE; however, limited discriminant validity, potential common method variance, and the predominantly video-based evaluation context warrant caution in interpreting these associations as distinct construct effects or as predictors of actual use. Qualitative findings from HCPs with direct clinical SAR exposure highlighted child-centered relational value and context-specific implementation concerns, suggesting that HCPs’ evaluations of patient-facing socially interactive technologies may involve considerations not fully represented by conventional user-centered technology acceptance constructs. Clinical utility, child-centered relational value, and conditions relevant to safe implementation therefore warrant further investigation. Multisite and longitudinal studies using validated measures and real-world SAR exposure are needed to examine how these considerations relate to actual uptake and sustained use across pediatric care contexts.

## Supplementary material

10.2196/105501 Multimedia Appendix 1 Qualitative and Quantitative data and measurement.

## Abbreviations

AVE: average variance extracted
BI: behavioral intention
EE: effort expectancy
FC: facilitating conditions
HCP: health care professional
HM: hedonic motivation
HTMT: heterotrait-monotrait ratio of correlations
IRB: Institutional Review Board
NASSS: Nonadoption, Abandonment, Scale-up, Spread, and Sustainability
PE: performance expectancy
PICU: pediatric intensive care unit
SAR: socially assistive robot
SI: social influence
TAM: Technology Acceptance Model
UTAUT2: Unified Theory of Acceptance and Use of Technology 2
VIF: variance inflation factor
