# Supplementary material for: Factors Associated With Pediatric Health Care Professionals’ Intention to Adopt Socially Assistive Robots: Mixed Methods Study
Source: J Med Internet Res. 2026 Oct 15;28:e105501. doi: 10.2196/105501 (PMC13671486; doi:10.2196/105501)
Supplement: Supplementary file 1 [file jmir-v28-e105501-s001.docx]

## Multimedia Appendix

### Table S1. Questionnaire items, constructs, and sources for the extended UTAUT2 instrument (24 items administered; 21 retained).

| **Construct** | **Item** | **Item Content** | **Source** | Corrected item-total correlation | Status |
| --- | --- | --- | --- | --- | --- |
| Performance Expectancy (PE) | Q1 | I believe SARs can improve my work efficiency. | Cannavacciuolo et al [1] | .638 | Retained |
|  | Q2 | I feel SARs can help me provide higher quality care. | Casas et al [2] | .696 | Retained |
|  | Q3 | I believe SARs are beneficial for patients. | Casas et al [2] | .573 | Retained |
|  | Q4¹ | I am concerned that if SARs malfunction, it will affect my daily work. (R)* | Raigoso et al [3] | .023 | Deleted |
| Effort Expectancy (EE) | Q5 | I feel I have the capability to use SARs. | Cannavacciuolo et al [1] | .427 | Retained |
|  | Q6 | Learning to operate SARs is easy for me. | Cannavacciuolo et al [1]; Raigoso et al [3] | .550 | Retained |
|  | Q7¹ | I find the operation of SARs difficult to understand. (R)* | Cannavacciuolo et al [1]; Casas et al [2] | −.130 | Deleted |
|  | Q8 | I am able to operate SARs independently without assistance. | Raigoso et al [3] | .404 | Retained |
| Social Influence (SI) | Q9 | I feel colleagues would support clinical use of SARs. | Raigoso et al [3] | .611 | Retained |
|  | Q10 | I feel supervisors would be willing to promote the use of SARs clinically. | Cannavacciuolo et al [1] | .705 | Retained |
|  | Q11 | I feel using SARs helps build a positive professional image. | Expert interviews | .631 | Retained |
| Facilitating Conditions (FC) | Q12 | I can obtain sufficient training resources to use SARs. | Cannavacciuolo et al [1] | .452 | Retained |
|  | Q13¹ | My work environment is not suitable for introducing SARs. (R)* | Expert interviews | .090 | Deleted |
|  | Q14 | I possess the knowledge required to use SARs. | Cannavacciuolo et al [1] | .270 | Retained |
| Hedonic Motivation (HM) | Q15 | I find using SARs enjoyable. | Cannavacciuolo et al [1] | .709 | Retained |
|  | Q16 | I do not think using SARs makes my work more interesting. (R)* | Casas et al [2] | .222 | Retained |
|  | Q17 | Using SARs clinically gives me a sense of accomplishment. | Cannavacciuolo et al [1] | .660 | Retained |
|  | Q18 | I look forward to each interaction experience with SARs. | Expert interviews | .714 | Retained |
| Trust (TR) | Q19 | I believe current SAR technology is sufficiently stable. | Cannavacciuolo et al [1] | .564 | Retained |
|  | Q20 | I can rely on SARs to help me complete certain work tasks. | Cannavacciuolo et al [1] | .566 | Retained |
|  | Q21 | I worry that SARs are prone to errors, affecting operational stability. (R)* | Cannavacciuolo et al [1] | .298 | Retained |
| Behavioral Intention (BI) | Q22 | I intend to continue using SARs clinically. | Cannavacciuolo et al [1] | .871 | Retained |
|  | Q23 | I would recommend that colleagues use SARs. | Cannavacciuolo et al [1] | .864 | Retained |
|  | Q24 | If SARs had more functions in the future, I would be more willing to use them. | Expert interviews | .746 | Retained |

Note. Superscript 1 denotes an item that was removed after item-level evaluation. R* = reverse-scored item.

### Table S2. Interview guide domains and sources (N=17). The full semi-structured interview guide used in the qualitative strand, including the source rationale for each domain.

| **No.** | **Interview Question** | **Source** |
| --- | --- | --- |
| 1 | When you observed a socially assistive robot accompanying children in the ward, how did you feel? | El-Gazar et al [4] |
| 2 | What are your thoughts on using socially assistive robots in pediatric settings? | Expert interviews |
| 3 | What technical or institutional challenges do you foresee in implementing socially assistive robots in practice? | El-Gazar et al [4]; Liang et al [5] |
| 4 | What factors influence your acceptance of socially assistive robots? | Rigaud et al [6]; Expert interviews |
| 5 | Under what clinical circumstances would you be more willing to use socially assistive robots in your work? | UTAUT2 theory; Bar-On et al [7] |
| 6 | Based on your clinical experience, what functions or features should socially assistive robots have to meet real-world clinical needs? | Expert interviews; El-Gazar et al [4] |
| 7 | Do you have any concerns or reservations about the clinical use of socially assistive robots? | El-Gazar et al [4] |
| 8 | Is there anything else you would like to share about the use of socially assistive robots? | Expert interviews |

### Table S3. Construct-level measurement properties of the final 21-item instrument: standardized factor loadings, composite reliability, and average variance extracted (N=125).

| **Construct** | **Item** | **Standardized loading (λ)** | **Composite reliability (ρc)** | **AVE** |
| --- | --- | --- | --- | --- |
| **PE** | Q1 | .898 | .881 | .714 |
|  | Q2 | .891 |  |  |
|  | Q3 | .729 |  |  |
| **EE** | Q5 | .868 | .795 | .573 |
|  | Q6 | .849 |  |  |
|  | Q8 | .530 |  |  |
| **SI** | Q9 | .826 | .813 | .593 |
|  | Q10 | .775 |  |  |
|  | Q11 | .706 |  |  |
| **FC** | Q12 | — | — | —^a^ |
|  | Q14 | — |  |  |
| **HM** | Q15 | .835 | .774 | .501 |
|  | Q16 | .227 |  |  |
|  | Q17 | .877 |  |  |
|  | Q18 | .764 |  |  |
| **TR** | Q19 | .762 | .708 | .476 |
|  | Q20 | .827 |  |  |
|  | Q21 | .333 |  |  |
| **BI** | Q22 | .884 | .916 | .785 |
|  | Q23 | .907 |  |  |
|  | Q24 | .868 |  |  |

*Note.*

ᵃ Composite reliability (ρc) and average variance extracted (AVE) are not calculated for the 2-item Facilitating Conditions subscale due to model under-identification in single-factor CFA; the Spearman-Brown coefficient (.662; inter-item correlation r = .495) is reported to evaluate reliability. Items Q16 and Q21 are reverse-coded. All standardized factor loadings are statistically significant at P < .001.

### Table S4. Heterotrait-monotrait ratios among study constructs (N=125).

| Construct | PE | EE | SI | FC | HM | TR | BI |
| --- | --- | --- | --- | --- | --- | --- | --- |
| PE | — |  |  |  |  |  |  |
| EE | .762 | — |  |  |  |  |  |
| SI | .789 | .812 | — |  |  |  |  |
| FC | .821 | .969 | .950 | — |  |  |  |
| HM | .794 | .805 | .936 | .882 | — |  |  |
| TR | .747 | .745 | .831 | .842 | .914 | — |  |
| BI | .791 | .768 | .862 | .833 | .906 | .824 | — |

Note. HTMT = heterotrait-monotrait ratio of correlations; PE = performance expectancy; EE = effort expectancy; SI = social influence; FC = facilitating conditions; HM = hedonic motivation; TR = trust; BI = behavioral intention.

### Table S5. Pearson correlations among UTAUT2 constructs and behavioral intention (N=125).

| **Construct** | **PE** | **EE** | | **SI** | **FC** | **HM** | **TR** | **BI** |
| --- | --- | --- | --- | --- | --- | --- | --- | --- |
| Performance Expectancy (PE) | — | |  |  |  |  |  | .729** |
| Effort Expectancy (EE) | .549** | | — |  |  |  |  | .550** |
| Social Influence (SI) | .712** | | .617** | — |  |  |  | .774** |
| Facilitating Conditions (FC) | .554** | | .700** | .690** | — |  |  | .567** |
| Hedonic Motivation (HM) | .640** | | .478** | .710** | .618** | — |  | .749** |
| Trust (TR) | .555** | | .525** | .580** | .578** | .624** | — | .627** |

Note. PE=Performance Expectancy; EE=Effort Expectancy; SI=Social Influence; FC=Facilitating Conditions; HM=Hedonic Motivation; TR=Trust; BI=Behavioral Intention. Diagonal entries are omitted because self-correlations equal 1.00. **P<.001 (2-tailed).

### Table S6. Exploratory analysis of prior clinical socially assistive robot exposure: descriptive statistics, nonparametric tests, effect sizes, sensitivity analysis, and supplementary parametric results (N=125).

| **Analysis** | **Behavioral intention** | **Overall acceptance** | **Note** |
| --- | --- | --- | --- |
| **Descriptive statistics by prior clinical SAR exposure group** | | | |
| No prior exposure (n=104), mean (SD) | 3.94 (0.62) | 3.70 (0.49) |  |
| No prior exposure (n=104), median [IQR] | 4.00 [3.67, 4.33] | 3.74 [3.37, 4.05] |  |
| 1 prior exposure (n=11), mean (SD) | 4.30 (0.64) | 4.13 (0.49) |  |
| 1 prior exposure (n=11), median [IQR] | 4.33 [4.00, 4.67] | 4.16 [3.89, 4.47] |  |
| ≥2 prior exposures (n=10), mean (SD) | 4.77 (0.52) | 4.44 (0.48) |  |
| ≥2 prior exposures (n=10), median [IQR] | 5.00 [4.67, 5.00] | 4.58 [4.16, 4.79] |  |
| **Primary omnibus analysis (nonparametric)** | | | |
| Kruskal-Wallis H (df=2) | 16.500 | 18.623 |  |
| P value | <.001 | <.001 |  |
| Epsilon-squared (ε²) | .133 | .150 | Omnibus effect size |
| **Pairwise comparisons (Mann-Whitney U; Bonferroni-adjusted α=.017)** | | | |
| None vs once: U (z) | 379.0 (−1.923) | 312.5 (−2.463) |  |
| None vs once: P | .055; adjusted P=.164 (ns) | .014; adjusted P=.041 |  |
| None vs once: effect size r | .179 | .230 | r=\|z\|/√N |
| None vs once: mean difference [95% CI] | +0.367 [−0.029, 0.736] | +0.434 [0.156, 0.744] | Bootstrap, 5000 resamples |
| None vs ≥2: U (z) | 163.0 (−3.708) | 148.0 (−3.729) |  |
| None vs ≥2: P | <.001; adjusted P<.001 | <.001; adjusted P<.001 |  |
| None vs ≥2: effect size r | .347 | .349 |  |
| None vs ≥2: mean difference [95% CI] | +0.831 [0.467, 1.100] | +0.741 [0.435, 1.028] |  |
| Once vs ≥2: U (z) | 32.0 (−1.725) | 34.5 (−1.409) |  |
| Once vs ≥2: P | .085; adjusted P=.254 (ns) | .159; adjusted P=.476 (ns) |  |
| Once vs ≥2: effect size r | .376 | .307 |  |
| Once vs ≥2: mean difference [95% CI] | +0.464 [−0.045, 0.933] | +0.308 [−0.102, 0.713] |  |
| **Sensitivity analysis: minimum detectable effect size (α=.017, 80% power)** | | | |
| None vs once: Cohen d | 1.04 | 1.04 | Determined by group sizes only |
| None vs ≥2: Cohen d | 1.08 | 1.08 |  |
| Once vs ≥2: Cohen d | 1.53 | 1.53 |  |
| **Supplementary parametric robustness analyses** | | | |
| One-way ANOVA F (2, 122) | 9.475 | 13.308 |  |
| ANOVA P value | <.001 | <.001 |  |
| Eta-squared (η²) | .134 | .179 |  |
| Welch F (2, 15.3) | 12.000 | 13.000 | Unequal variances |
| Welch P value | <.001 | <.001 |  |

Note. SAR=socially assistive robot; ns=not significant; IQR=interquartile range.

Prior clinical SAR exposure refers to previous exposure to the physical SAR in the clinical setting; exposure to the standardized prerecorded video was not included. Overall acceptance was defined as the mean of the 6 predictor constructs (PE, EE, SI, FC, HM, and TR), excluding behavioral intention. Epsilon-squared was calculated as H/[(N²−1)/(N+1)]. Pairwise P values are reported unadjusted and were evaluated against a Bonferroni-adjusted threshold of α=.017; Bonferroni-adjusted P values (unadjusted P multiplied by 3) are also shown. Pairwise effect sizes were calculated as r=|z|/√N, where N is the combined size of the 2 groups being compared. Mean differences are later group minus earlier group, with bias-corrected bootstrap 95% CIs based on 5000 resamples. Kruskal-Wallis and Mann-Whitney U tests were treated as the primary analyses for these comparisons; the one-way ANOVA and Welch tests are reported only as robustness checks. The exposure groups were small and unequal and the study was not powered for these subgroup contrasts: at α=.017 and 80% power, only very large standardized differences were detectable. All results in this table are exploratory, descriptive, and hypothesis-generating and should not be interpreted as evidence of an exposure-response relationship.

### Table S7. Comparison of physicians and nurses on UTAUT2 constructs, behavioral intention, and overall acceptance (N=125).

| Construct | Physicians M (SD) | Nurse M (SD) | t | p | Cohen's d |
| --- | --- | --- | --- | --- | --- |
| PE | 4.08 (0.63) | 4.14 (0.66) | −0.43 | .667 | −0.10 |
| EE | 3.79 (0.65) | 3.78 (0.68) | 0.07 | .947 | 0.02 |
| SI | 4.03 (0.54) | 4.04 (0.60) | −0.10 | .919 | −0.02 |
| FC | 3.58 (0.69) | 3.79 (0.72) | −1.32 | .190 | −0.30 |
| HM | 3.68 (0.59) | 3.75 (0.65) | −0.46 | .648 | −0.10 |
| TR | 3.35 (0.57) | 3.35 (0.68) | −0.05 | .964 | −0.01 |
| BI | 4.04 (0.68) | 4.03 (0.65) | 0.05 | .964 | 0.01 |
| Acceptance | 3.75 (0.46) | 3.81 (0.55) | −0.48 | .630 | −0.11 |

### Table S8. Comparison of clinical units on UTAUT2 constructs, behavioral intention, and overall acceptance (N=125).

| **Construct** | **Pediatric Internal Medicine**  **(n=44)**  **M (SD)** | **PICU**  **(n=39)**  **M (SD)** | **Pediatric Emergency**  **(n=31)**  **M (SD)** | **Pediatric Surgery**  **(n=11)**  **M (SD)** | **F (3, 121)** | **p** | **η²** | **Sig.** |
| --- | --- | --- | --- | --- | --- | --- | --- | --- |
| Performance Expectancy (PE) | 4.17 (0.61) | 3.97 (0.70) | 4.23 (0.59) | 4.27 (0.77) | 1.291 | .281 | — | ns |
| Effort Expectancy (EE) | 3.83 (0.68) | 3.67 (0.64) | 3.73 (0.67) | 4.09 (0.76) | 1.301 | .277 | — | ns |
| Social Influence (SI) | 4.06 (0.57) | 3.85 (0.58) | 4.11 (0.53) | 4.39 (0.68) | 2.945 | .036 | .068 | * |
| Facilitating Conditions (FC) | 3.70 (0.68) | 3.58 (0.72) | 3.81 (0.64) | 4.36 (0.74) | 3.837 | .012 | .087 | * |
| Hedonic Motivation (HM) | 3.70 (0.60) | 3.50 (0.54) | 3.87 (0.64) | 4.27 (0.71) | 5.473 | .002 | .119 | ** |
| Trust (TR) | 3.41 (0.55) | 3.01 (0.58) | 3.51 (0.60) | 3.91 (0.91) | 7.973 | <.001 | .165 | *** |
| Behavioral Intention (BI) | 4.12 (0.67) | 3.79 (0.58) | 4.09 (0.62) | 4.42 (0.70) | 3.707 | .014 | .084 | * |
| Overall Acceptance | 3.81 (0.48) | 3.60 (0.50) | 3.87 (0.49) | 4.22 (0.71) | 4.715 | .004 | .105 | ** |
| **Bonferroni-adjusted post hoc pairwise comparisons (α=.0083 for 6 pairwise contrasts)** | | | | | | | | |
| Performance Expectancy (PE) | internal medicine vs PICU, P=.892; internal medicine vs emergency, P=1.000; internal medicine vs surgery, P=1.000; PICU vs emergency, P=.601; PICU vs surgery, P=1.000; emergency vs surgery, P=1.000 | | | | | | | |
| Effort Expectancy (EE) | internal medicine vs PICU, P=1.000; internal medicine vs emergency, P=1.000; internal medicine vs surgery, P=.402; PICU vs emergency, P=1.000; PICU vs surgery, P=1.000; emergency vs surgery, P=.779 | | | | | | | |
| Social Influence (SI) | internal medicine vs PICU, P=.621; internal medicine vs emergency, P=1.000; internal medicine vs surgery, P=.514; PICU vs emergency, P=.408; PICU vs surgery, P=.039; emergency vs surgery, P=.931 | | | | | | | |
| Facilitating Conditions (FC) | internal medicine vs PICU, P=1.000; internal medicine vs emergency, P=1.000; internal medicine vs surgery, P=.033; PICU vs emergency, P=1.000; PICU vs surgery, P=.007; emergency vs surgery, P=.140 | | | | | | | |
| Hedonic Motivation (HM) | internal medicine vs PICU, P=.749; internal medicine vs emergency, P=1.000; internal medicine vs surgery, P=.036; PICU vs emergency, P=.070; PICU vs surgery, P=.002; emergency vs surgery, P=.357 | | | | | | | |
| Trust (TR) | internal medicine vs PICU, P=.020; internal medicine vs emergency, P=1.000; internal medicine vs surgery, P=.099; PICU vs emergency, P=.006; PICU vs surgery, P<.001; emergency vs surgery, P=.369 | | | | | | | |
| Behavioral Intention (BI) | internal medicine vs PICU, P=.108; internal medicine vs emergency, P=1.000; internal medicine vs surgery, P=.957; PICU vs emergency, P=.313; PICU vs surgery, P=.023; emergency vs surgery, P=.791 | | | | | | | |
| Overall Acceptance | internal medicine vs PICU, P=.328; internal medicine vs emergency, P=1.000; internal medicine vs surgery, P=.129; PICU vs emergency, P=.154; PICU vs surgery, P=.003; emergency vs surgery, P=.357 | | | | | | | |
| **Minimum detectable effect size for pairwise contrasts (Cohen d; α=.0083, 80% power)** | | | | | | | | |
| Pediatric internal medicine vs PICU | 0.78 | | | | | | | |
| Pediatric internal medicine vs pediatric emergency | 0.84 | | | | | | | |
| Pediatric internal medicine vs pediatric surgery | 1.21 | | | | | | | |
| PICU vs pediatric emergency | 0.86 | | | | | | | |
| PICU vs pediatric surgery | 1.23 | | | | | | | |
| Pediatric emergency vs pediatric surgery | 1.28 | | | | | | | |
| **Exploratory contrast: PICU (n=39) versus all other units combined (n=86)** | | | | | | | | |
| Trust (TR) | 3.01 vs 3.51; t123=−4.18, P<.001, Cohen d=−0.81 | | | | | | | |
| Hedonic Motivation (HM) | 3.50 vs 3.84; t123=−2.83, P=.005, Cohen d=−0.55 | | | | | | | |
| Behavioral Intention (BI) | 3.79 vs 4.15; t123=−2.94, P=.004, Cohen d=−0.57 | | | | | | | |
| Overall Acceptance | 3.60 vs 3.88; t123=−2.91, P=.004, Cohen d=−0.56 | | | | | | | |

Note. M = mean on 5-point Likert scale; SD = standard deviation; PICU = pediatric intensive care unit; η² = eta-squared. All construct scores were calculated using the final 21-item instrument. FC was calculated from the retained 2-item subscale (Q12 and Q14), and overall acceptance was defined as the mean of the 6 predictor constructs (PE, EE, SI, FC, HM, and TR), excluding BI. ns = not significant In the post hoc panel, unit names are abbreviated (internal medicine = pediatric internal medicine; emergency = pediatric emergency; surgery = pediatric surgery), and all pairwise P values are Bonferroni-adjusted across the 6 unit contrasts within each construct. These pairwise contrasts were exploratory and were not prespecified; the minimum detectable effect sizes shown below indicate that they were powered to detect only large standardized differences.; — = η² not computed for non-significant results. *P<.05; **P<.01; ***P<.001.

### Table S9. Condensed thematic coding framework derived from semistructured interviews with pediatric health care professionals (N=17).

| **#** | **Theme** | **Categories** |
| --- | --- | --- |
| **1** | **Emotional and Stress-Related Challenges of Hospitalized Children** | (1) Stress responses and reactions in hospitalized contexts  (2) Physician–patient relationship tensions  (3) Caregiver–provider cognitive discrepancies  (4) Clinical professional role demands and workload  (5) Developmental-stage differences in children’s expression of distress and coping |
| **2** | **Functional Roles and Clinical Positioning of SARs** | (1) Companionship and interactive support in pediatric care  (2) Routine repetitive tasks and health education  (3) Clinical care assistance  (4) Automated monitoring, real-time alerting, and documentation  (5) Health-promoting and rehabilitation behavior support  (6) Psychological support for nursing staff |
| **3** | **Health Care Providers’ Attitudes Toward SARs** | (1) Facilitating factors  (2) Barriers  (3) Perceptions and attitudes toward SARs  (4) Anticipated reactions from children and families |
| **4** | **Child-Centered Design Considerations** | (1) Height and child-level perspectives  (2) SAR appearance and functional design  (3) Multimodal interaction feature design  (4) Autonomy and ease-of-operation considerations |
| **5** | **Practical Considerations for Clinical Implementation** | (1) Technological stability and equipment reliability  (2) Clinical safety—physical, psychological, and infection control  (3) Clinical environmental constraints  (4) Usage acceptance and trust issues  (5) SAR functionality and quantity configuration  (6) Information accuracy and stability |
| **6** | **Institutional Decision-Making and Management** | (1) Medical liability attribution  (2) Information security and privacy protection  (3) Cost-effectiveness and equipment management  (4) Administrative systems and governance  (5) External and institutional influences on adoption decisions  (6) Education and training |

### Table S10. Mixed methods joint display of convergence, complementarity, and divergence across the quantitative (N=125) and qualitative (N=17) strands.

| **Type** | **Core Finding** | **Quantitative Evidence** | **Qualitative Evidence** | **Integrated Interpretation** |
| --- | --- | --- | --- | --- |
| **Convergence** | Performance expectancy and perceived clinical utility align | PE was positively associated with BI (β=.238, *P*=.001). | Participants described SARs as useful for patient education, distraction, companionship, communication, and repetitive supportive activities. | The quantitative and qualitative findings converged on perceived clinical utility as a relevant consideration in HCPs’ evaluations of SARs. |
| **Complementarity** | Trust-related concerns contextualized the quantitative TR finding | TR showed a small association with BI that was sensitive to model specification, and its measurement properties indicated limited convergent and discriminant validity. | Participants emphasized reliability, information accuracy, privacy, infection control, maintenance, and accountability. | Qualitative accounts provided contextual detail regarding trust-related concerns, particularly reliability, information accuracy, privacy, and accountability. These findings should not be interpreted as evidence of a robust independent quantitative effect of TR. |
| **Complementarity** | PICU participants expressed heightened trust-related caution | PICU participants reported lower TR than participants from other units; however, the TR×PICU interaction was not statistically significant. | PICU participants described low tolerance for errors in high-acuity care. | Qualitative accounts suggested greater assurance requirements in high-acuity care, but the quantitative data did not support a moderation effect by PICU setting. |
| **Divergence** | HCP-centered hedonic motivation and child-centered relational value had different referents | HM was positively associated with BI, but limited discriminant validity warrants caution in interpreting HM as a distinct construct-specific effect. | Interviewees focused on children’s engagement, comfort, distraction, and companionship rather than HCP enjoyment. | The quantitative HM construct reflected HCPs’ own enjoyment, whereas the qualitative accounts focused primarily on children’s enjoyment, comfort, and engagement. Child-centered relational value should therefore not be used to explain or validate the quantitative HM association. |
| **Divergence** | Operational burden was salient qualitatively but EE was nonsignificant | EE was not significantly associated with BI in the adjusted model. | Participants raised activation burden, workflow fit, maintenance, and supervision concerns. | The quantitative EE construct reflected perceived personal ease or capability, whereas qualitative concerns extended to workflow compatibility, training, maintenance, and broader implementation burden. The nonsignificant EE association should not be interpreted as evidence that usability or operational considerations are unimportant. |
| **Divergence** | Social influence was prominent quantitatively but was not articulated as a salient consideration qualitatively | SI had the largest standardized coefficient in the adjusted model (β=.348, P<.001), although limited discriminant validity warrants caution in construct-specific interpretation. | Peer and supervisory expectations were not articulated by participants as bearing on their willingness to use SARs. | The prominence of SI in the quantitative model was not mirrored in the interviews. Its psychological meaning therefore remains uncertain and should not be interpreted as evidence of a clearly defined normative, institutional, or cultural mechanism. |
| **Exploratory** | Exploratory association with prior clinical SAR exposure | Kruskal-Wallis tests indicated differences across prior clinical SAR exposure groups for BI (H2=16.50, P<.001) and overall acceptance (H2=18.62, P<.001). The exposed subgroups were small and unequal (1 previous exposure, n=11; ≥2 previous exposures, n=10). | Participants described direct exposure as useful for judging SAR usability and clinical relevance. | Higher BI and overall acceptance were observed with greater prior clinical SAR exposure, but the small, unequal, and underpowered subgroups preclude causal or exposure-response interpretations. These findings are descriptive and hypothesis-generating. |

### Figure S1. Parallel analysis scree plot for the final 21-item instrument.

Note. Parallel analysis was conducted as an exploratory dimensionality check. The scree plot shows a dominant first factor and does not support retaining a seven-factor solution in this sample. This finding was treated as a measurement limitation rather than used to redefine the theory-derived UTAUT2 constructs, and construct-specific associations were interpreted accordingly.

## Reference

1. Cannavacciuolo L, Rippa P, Caputo S. User acceptance of healthcare robots through extended UTAUT2: a mixed method approach. Research Square. Preprint posted online 2022. [doi: 10.21203/rs.3.rs-2326113/v1]

2. Casas JA, Céspedes N, Cifuentes CA, Gutierrez LF, Rincón-Roncancio M, Múnera M. Expectation vs reality: attitudes towards a socially assistive robot in cardiac rehabilitation. Appl Sci. 2019;9(21):4651. [doi: 10.3390/app9214651]

3. Raigoso D, Céspedes N, Cifuentes CA, Del-Ama AJ, Múnera M. A survey on socially assistive robotics: clinicians’ and patients’ perception of a social robot within gait rehabilitation therapies. Brain Sci. 2021;11(6):738. [doi: 10.3390/brainsci11060738]

4. El-Gazar HE, Abdelhafez S, Ali AM, Shawer M, Alharbi TAF, Zoromba MA. Are nurses and patients willing to work with service robots in healthcare? A mixed-methods study. BMC Nurs. 2024;23(1):718. [doi: 10.1186/s12912-024-02336-7]

5. Liang HF, Wu KM, Weng CH, Hsieh HW. Nurses’ views on the potential use of robots in the pediatric unit. J Pediatr Nurs. 2019;47:e58-e64. [doi: 10.1016/j.pedn.2019.04.027]

6. Rigaud AS, Dacunha S, Harzo C, Lenoir H, Sfeir I, Piccoli M, et al. Implementation of socially assistive robots in geriatric care institutions: healthcare professionals’ perspectives and identification of facilitating factors and barriers. J Rehabil Assist Technol Eng. 2024;11:20556683241284765. [doi: 10.1177/20556683241284765]

7. Bar-On I, Mayo G, Levy-Tzedek S. Socially assistive robots for Parkinson’s disease: needs, attitudes and specific applications as identified by healthcare professionals. ACM Trans Hum-Robot Interact. 2023;12(1):11. [doi: 10.1145/3570168]
